# Sharing behavior and health care utilization following direct-to-consumer genetic testing: a systematic review

**DOI:** 10.12688/openreseurope.19751.1

**Published:** 2025-03-21

**Authors:** Eva Van Steijvoort, Kaatje Goossens, Kenji Demesure, Alexandra Stanczak, Maria Siermann, Pascal Borry

**Affiliations:** 1 Department of Public Health and Primary Care, Centre for Biomedical Ethics and Law, KU Leuven, Leuven, 3000, Belgium; 2 Department of Health, Ethics & Society, Maastricht University GROW School for Oncology and Reproduction, Maastricht, Limburg, The Netherlands

**Keywords:** Genetic testing, Direct to consumer, Public Health, Prevention, Genomics, Screening, Predictive Testing

## Abstract

**Background:**

Direct-to-consumer genetic testing (DTC-GT) which provides genetic information directly to the public, has become widely available at a moderate cost. Since DTC-GT companies frequently recommend that consumers consult healthcare professionals for assistance in interpreting and using genetic health risk information, this could potentially have an impact on healthcare systems.

**Methods:**

We performed a systematic review to assess: (1) the sharing behavior of actual DTC-GT consumers, (2) experiences of healthcare professionals regarding DTC-GT consumers sharing their test results and (3) healthcare utilization following DTC-GT, with a particular focus on validation of DTC-GT results and subsequent clinical actions. Our systematic review was registered in PROSPERO under the registration number CRD42024517079.

**Results:**

Our search identified 40 unique articles eligible for inclusion that were published between 2009 en 2022. The proportion of participants who shared their DTC-GT test results with a health care professional ranged from 1% to 57%. DTC-GT consumers most commonly reported sharing their results with a primary healthcare professional. The proportion of health care professionals that had experiences with DTC-consumers sharing their test results ranged from 19% to 76%. The percentage of participants sharing their DTC-GT test with family members ranged from 18% to 98%. More detailed analysis indicated that this was frequently the case with partners, parents, and siblings. Sharing of test results with extended family members occurred less frequently. Several studies reported on instances of DTC-GT result validation and clinical actions performed based on the DTC-GT findings

**Conclusion:**

While initial concerns about the impact of DTC-GT on health care systems have not fully materialized, the increasing number of consumers consulting with healthcare professionals underscores the need for preparedness and appropriate policy responses. Future research should prioritize standardizing study methodologies and expanding investigations beyond the U.S. context to better capture the global impact of DTC-GT.

## Plain language summary

Direct-to-consumer genetic testing (DTC-GT) is now widely available, allowing people to learn about their genetic health risks without the involvement of a health care professional. Many companies offering these tests recommend that consumers talk to healthcare professionals to help understand their results, which could affect the healthcare system. We reviewed studies to learn about three things: (1) how frequently people share their genetic test results after using a DTC-GT, (2) healthcare professionals’ experiences with consumers sharing their DTC-GT results, and (3) how people use healthcare services after taking a DTC-GT, particularly in confirming results and making health decisions based on them. We found 40 relevant studies published between 2009 and 2022. The number of people who shared their genetic test results with a healthcare professional varied, ranging from 1% to 57%. Most often, people shared their results with their primary healthcare provider. Healthcare professionals had experience with consumers sharing their genetic test results in 19% to 76% of cases. Many consumers also shared their results with family members, with a range of 18% to 98% sharing their results, mostly with close family like partners, parents, and siblings. Fewer people shared their results with extended family. Some studies also mentioned that healthcare professionals sometimes confirmed the test results and took action based on them. Even though initial worries about the impact of DTC-GT on healthcare systems has not fully happened yet, our results show that an increasing number of people that use DTC-GT are consulting healthcare professionals. This shows a growing need for healthcare systems to have appropriate policies in place. Future research should focus on creating consistent study methods and look at how DTC-GT is affecting healthcare worldwide, beyond just the U.S.

## Introduction

The field of genetic and genomic medicine, considered to hold substantial potential for personalized medicine, is increasingly evolving from primarily focusing on rare monogenic conditions to the prediction of risk for prevalent complex conditions ^1, 2^. Providing information on predispositions for specific diseases or conditions is seen as a way forward to empower citizens and patients, enabling them to proactively manage their health and potentially prevent the (early) onset of certain conditions ^3^. The utilization of genetic information to inform lifestyle changes aimed at improving health outcomes, has also captured the attention of private companies. Over the past two decades we have witnessed the emergence of several models to provide genetic testing information directly to the public. Commercial laboratories have been increasingly marketing and selling a wide range of genetic tests online, allowing consumers to purchase them directly without requiring a healthcare professional’s involvement or prescription ^4^. The online format of DTC-GT quickly gained popularity due to its accessibility and affordability ^3, 5^.

The model of direct-to-consumer genetic testing (DTC-GT) deviates from the traditional provision of genetic testing information, in which a healthcare professional is responsible for ordering, testing, interpreting, and communicating genetic testing results ^4, 6^. In the DTC-GT model, consumers usually order a test kit online after approving terms of services. Upon receiving a test kit at home, consumers are instructed to produce a saliva sample and to send it to the DTC-GT company for genetic analysis. Once the analysis is complete, test results are directly delivered to the consumer through a personalized online platform or mobile application ^7, 8^. In the context of DTC-GT, test offerings are generally categorized into health-related and non-health-related types. Non-health-related DTC-GT tests include services such as ancestry testing, paternity testing, and assessments of specific traits (e.g. earwax type). Health-related DTC-GT, on the other hand, encompass various types of testing, including susceptibility testing for polygenic and multifactorial conditions (e.g. cardiovascular disease, type 2 diabetes), carrier screening for autosomal recessive conditions (e.g. cystic fibrosis) and/or X-linked conditions (e.g. Fragile X syndrome) to inform reproductive decision making, and pharmacogenomic tests to provide insights into personal drug responses ^9^. Differentiating between health and non-health-related DTC-GT offers can however be complex, particularly with the availability of third-party interpretation services. These services allow consumers to analyze their uninterpreted raw genetic data. As a result, they may also report certain variants and risk scores that fall outside the scope of the DTC-GT test that was originally purchased ^3, 10, 11^. Therefore, these services blur the line between genetic health risk information and non-health-related DTC-GT products, which adds complexity to regulatory efforts ^12, 13^.

DTC-GT offers that provide health information are currently widely available in many countries at a moderate cost ^2, 5^. As of 2023, 23andMe – a prominent player in the field – has a customer base exceeding 14 million genotyped customers ^14^. Surveys in the USA and Australia found that the majority (77% and 65% respectively) of those with experience with genetic testing were consumers of DTC-GT ^15, 16^. While the exact number of consumers in Europe remains unknown to date, the uptake of DTC-GT is also believed to be substantial ^4^. The global DTC-GT market is expected to further grow and to be worth over $4 billion by 2025 because of advances in technology, increased consumer demand and the desire for personalized health management ^3, 17, 18^. The rapid growth of the DTC-GT market has however raised several concerns with regard to the privacy and security of genetic data, as well as the accuracy/validity of the test results ^3, 19^. The reliability of a positive test or increased risk result after DTC-GT remains limited, as the development of many genetic conditions is also influenced by other factors such as the environment and lifestyle choices. The majority of DTC-GT also do not sequence the entire genome. Instead, they often use SNP-chip genotyping, a method examining the presence or absence of specific variants in the genetic code, including single nucleotide polymorphisms (SNPs) or small insertions or deletions. While SNP-chip genotyping is effective in detecting common genetic variants, it tends to have a higher error rate when analyzing very rare variants, which can lead to an increased likelihood of false positives ^11^.

More recently, some commercial companies have also started to offer whole genome or exome sequencing and the return of raw genomic data without interpretation ^4, 11^. These tests sequence nearly the entire genetic code and identify the genetic variants within it. The capability to predict disease risk through whole genome or exome sequencing data might motivate an increasing number of individuals to explore genomic technologies for personal health risk prediction ^20^. Yet even though data obtained from whole genome or exome sequencing can be valuable for understanding rare conditions in specific individuals, there is currently inadequate evidence to prove that expanding this technology to the general population would result in considerable benefits ^21, 22^. Interpretation of genetic variants is still challenging and largely depends on context ^11^. The predictive significance of a "disease-causing variant" is often substantially diminished when identified without the presence of a family history linked to the relevant condition ^23^. To date, the scientific understanding of genomic sequencing data remains incomplete, creating uncertainties about the real added value of this extensive information.

Since the emergence of DTC-GT, stakeholders have voiced and debated several potential risks and benefits ^1^. A particular concern has been the potential impact of DTC-GT on healthcare systems, particularly in terms of downstream effects, such as an increased demand for specialist consultations and follow-up testing. Since DTC-GT companies frequently recommend that consumers consult healthcare professionals for assistance in interpreting and using genetic health risk information, this could potentially have an impact on healthcare systems. Healthcare professionals have also voiced that they feel obligated to refer patients to specialists or suggest additional screening procedures based on DTC-GT results ^24^. A previous systematic literature review by Stewart *et al.* (2018), which covered studies up to January 2017, found that one-third of participants shared their results with at least one healthcare professional, often leading to additional follow-up tests ^25^. This review, however, specifically focused on sharing behavior and health care utilization following multiplex genetic testing, excluding tests related to nutrigenetics, pharmacogenetics, sports genetics, as well as those for highly penetrant genes and prenatal or neonatal testing ^25^. Furthermore, Stewart *et al.* (2018) focused solely on the sharing behavior of DTC-GT consumers and did not include any findings on the experiences of healthcare professionals regarding DTC-GT consumers sharing their test results ^25^. Given that these earlier research findings also largely reflect the sharing behavior and health care utilization of early adopters of DTC-GT and may no longer represent the current population undergoing DTC-GT due to the rapid growth of the industry, we aim to provide an updated overview based on the current available evidence. Therefore, the goal of this systematic review was to assess: (1) the sharing behavior of actual DTC-GT consumers, (2) experiences of healthcare professionals regarding DTC-GT consumers sharing their test results and (3) healthcare utilization following DTC-GT, with a particular focus on validation of DTC-GT results and subsequent clinical actions.

## Methods

This systematic review was registered in PROSPERO under the registration number CRD42024517079.

### Design and search strategy

**Figure 1.**
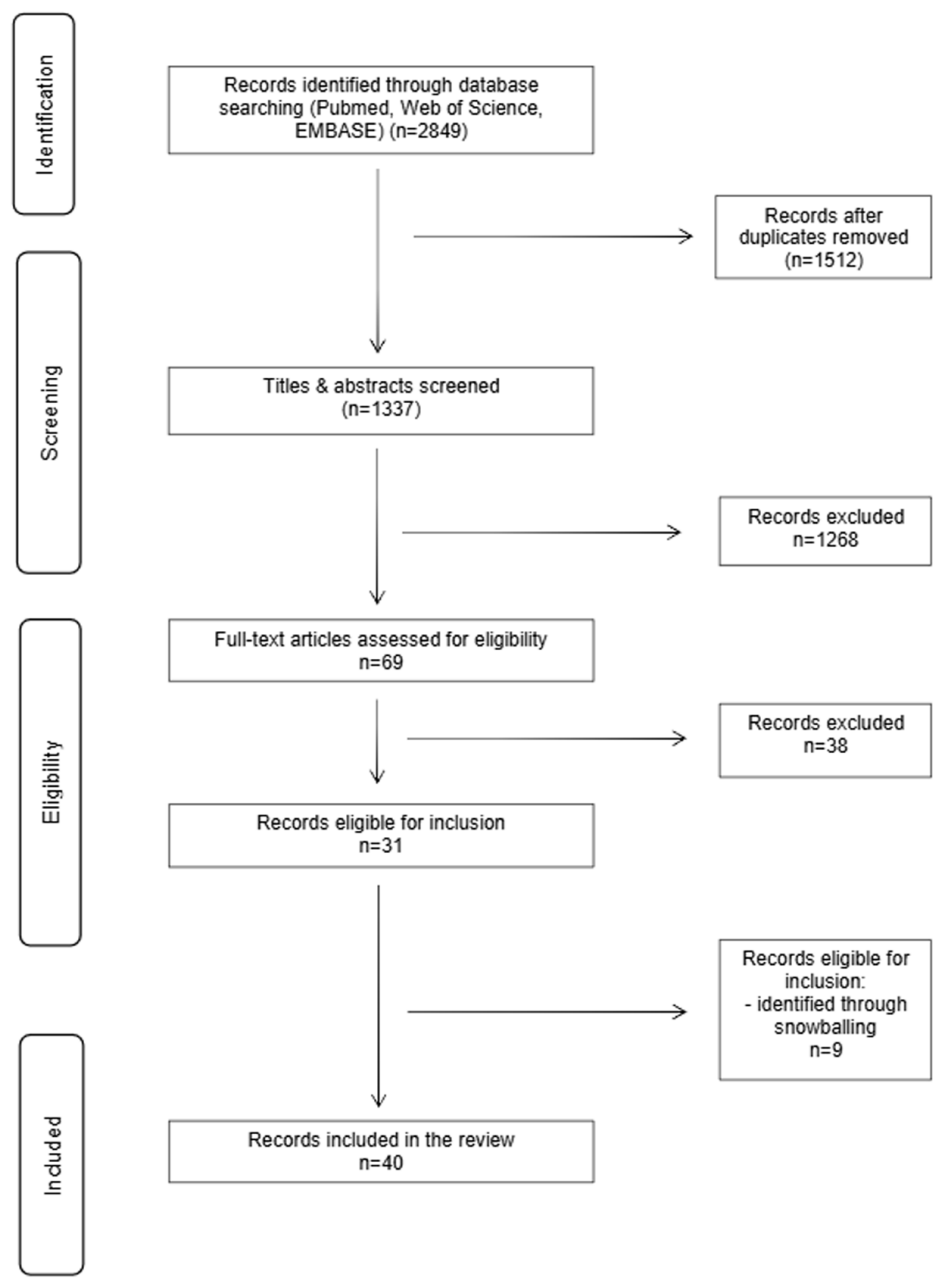
Graphically summarizes the literature search process in accordance with the PRISMA guidelines ^26^.

Our review process consisted of three steps. First, we performed a systematic literature search to identify relevant publications in three different databases: Web of Science, PubMed, and EMBASE. No restrictions on the publication date were applied during the initial search. On January 2 ^nd^ 2024, we made use of the following search string: ("Direct-to-consumer genomics" OR "Direct-to-consumer genetic testing" OR "direct-to-consumer personal genomic testing" OR "personal genetic testing" OR "personal genomics" OR "consumer genomics" OR "personal genomic testing" OR "Direct Access Genetic Testing" OR "personal genetics" OR "Direct-to-consumer genome scanning" OR "Direct-to-Consumer genome wide Profiling"). In a second phase, we consulted references from relevant papers to find any additional publications warranting inclusion (i.e. snowball method). Our review followed PRISMA guidelines for systematic reviews ^26^. ( Figure 1) Initially, all records identified were screened based on their title and abstract by two individual researchers (EVS and KD). After the exclusion of non-relevant records, the same two researchers (EVS and KD) independently assessed the full texts of the remaining records. Any disagreements during the selection procedure were discussed until consensus was achieved.

### Inclusion and exclusion criteria

Studies were eligible for inclusion if they were published in English and provided empirical research findings. Studies reporting on non-empirical work (e.g. case studies, commentaries, review, books, guidelines, etc.) were excluded. Only studies that evaluated the sharing behavior of DTC-GT consumers after they had received their test results were eligible for inclusion. Actual DTC-GT consumers were defined as individuals who either paid for DTC-GT out of pocket or were offered DTC-GT at a subsidized rate or for free in a research context. Studies that assessed the willingness to share DTC-GT results prior to obtaining the test results or in a hypothetical context (non-actual DTC-GT consumers) were excluded from this review. Likewise, we also excluded studies that reported on health care professionals being asked questions by patients about DTC-GT before undergoing the test. When a single research project was reported in multiple publications (evaluation of sharing behavior at multiple time points), we included the publication that evaluated sharing behavior at the most recent time point. When the data on the desired outcomes were not clearly reported in the article or accessible as supplementary materials, we reached out to the corresponding authors of the articles to inquire about the possibility of obtaining the data of interest.

### Data extraction

Data extraction was carried out by KD, KG, AS and checked for consistency by EVS. Any discrepancies were resolved through discussion until a consensus was reached. All data were extracted by using an extraction form that was created with Excel. In the first round of data extraction, we assessed study characteristics by focusing on the following variables for studies examining the sharing behavior of DTC-GT consumers: first author, year of publication, country of the research team, study name (if applicable), research methods, key demographic characteristics of study participants, information on which DTC-GT product/service participants had used, actual or hypothetical DTC-GT consumers, whether sharing behavior of DTC-GT consumers was assessed before or after receiving test results and whether DTC-GT consumers paid out-of-pocket or were offered DTC-GT at a subsidized rate or for free in a research context. In addition, the following information was sought for those studies that explored the experiences of different health care professionals with DTC-GT consumers sharing their test results: first author, year of publication, country of the research team, research methods, type of health care professionals. In the second phase, we extracted data about the sharing behavior of DTC-GT consumers. For this, we focused on whether DTC-GT consumers shared their test results with health care professionals (including primary care professional; genetic counselor; clinical geneticist, and other healthcare professionals) or family members (such as spouse, parents, siblings, children, and other extended family members). Other healthcare professionals (e.g. oncologist, clinical nurse) were grouped into a single overarching category. Extended family members were defined to include aunts, uncles, cousins or grandparents. Within the studies that explored the experiences of health care professionals with DTC-GT consumers sharing their test results we focused on whether participants had patients sharing DTC-GT with them and the number of patients who brought in results from DTC-GT. In a final stage, we examined healthcare utilization following DTC-GT. We specifically investigated whether results from DTC-GT were validated through further testing and whether any subsequent clinical actions were performed based on the DTC-GT findings.

### Quality appraisal

We performed an indicative quality appraisal of the included primary studies using the tool developed by Hawker *et al.* (2002) ^27^. Articles were not excluded from our systematic review based on their methodological quality. The quality appraisal was performed independently by two researchers (KG & AS) and checked for consistency by MS. In case of disagreement, the specific item was discussed until mutual agreement was reached.

## Results

### I. Search outcomes

Our database search led to the identification of 2849 articles. After the removal of duplicates (n=1512), we proceeded to the screening of 1337 records. Based on an evaluation of titles and abstracts, 1268 records were excluded. Subsequently, 69 full-text articles were assessed for eligibility. Of these, 38 articles were excluded for the following reasons: not reporting empirical findings or outcomes of interest, only reporting hypothetical sharing behavior, publications from a research project that resulted in multiple publications or data on outcomes of interest were not clearly reported and/or available (n=2 ( 15, 28)); despite efforts to reach corresponding authors). After consulting the references of the remaining 31 records that were eligible for inclusion, we identified 9 more relevant papers warranting inclusion. In total, 40 unique articles were included for the final analysis.

### II. Quality appraisal

The detailed findings of the quality assessment can be found in the supplementary information (see extended data).

### III. Study characteristics

A detailed overview of the primary empirical studies included in this systematic review is presented in Tables 1 and 2 (underlying data). The publication range of the 40 unique studies eligible for inclusion dates from 2007 to 2022. The majority (n=28/40) ^29– 56^ of studies assessed the sharing behavior of DTC-GT consumers (see Table 1) (underlying data), while 13 studies ^29, 57– 68^ reported on the experiences of health care professionals with DTC-GT consumers sharing their test results (see Table 2 underlying data). Almost 90% of the identified records originated from research projects that were carried out by research teams based in the USA (n=35/40) ^15, 28– 47, 49, 50, 52– 55, 57– 59, 61, 63, 64, 66– 71^. Most studies used quantitative research methods (n=33/40) ^29, 30, 32– 35, 37, 38, 40– 47, 49, 50, 52– 68^, whereas a smaller number used qualitative (n=3/40) ^31, 36, 39^ or mixed methods approaches (n=4/40) ^48, 49, 51, 68^. Most articles (n=21/28) included study participants who had paid out-of-pocket for their DTC-GT ^29, 30, 33, 36, 37, 40– 55^. In contrast, three studies had provided a DTC-GT for free to study participants ^31, 32, 39^ and ten had offered a DTC-GT at a subsidized rate as part of a research project ^34, 35, 38, 40– 42, 44– 46, 56^. Within the studies assessing sharing behavior of DTC-GT consumers, six articles were the result of the ‘Impact of Personal Genomics (PGen)’ where consumers of two DTC-GT companies (23andMe and Pathway Genomics) based in the USA were surveyed ^40– 42, 44– 46^ and three articles resulted from the American ‘Scripps Genomic Health Initiative (SGHI)’, which was a research project where study participants received the Navigenics Health Compass at a subsidized rate ^34, 35, 38^. An overview of key characteristics of study participants from the primary studies included in this review is available as in Table 1 and 2 (Extended data).

### IV. Main findings

***A. Sharing behavior***

**Sharing behavior of DTC-GT consumers with health care professionals**

A total of 28 studies reported study findings of research projects that had assessed the extent to which DTC-GT consumers shared their genetic test results with healthcare professionals, with the number of participants included in the studies ranging from 20 to 30385 (see Table 3A) (underlying data) ^29– 56^. The proportion of participants who shared their DTC-GT test results with a health care professional ranged from 1% ^32^ to 57% ^37^. Overall, the rate of sharing appears to have remained relatively stable over the years. Between 2007 and 2011, the sharing rate ranged from 10% to 53% ^29, 30^. From 2012 to 2016, it fluctuated from 1% to 57% ^31– 33, 37, 40– 44^, and between 2017 and 2022, it ranged from 6% to 45% ^45, 47– 50, 53– 55^. Among participants who paid out of pocket, the sharing rate ranged from 6% to 57% ^29, 30, 33, 37, 43, 47– 50, 53– 55^. For those who received a subsidized test, the sharing rate was between 19% and 39% ^34, 35, 38, 40– 42, 44– 46, 56^, while participants who received a free test shared their results at a rate ranging from 1% to 42% ^31, 32, 39^.

**Figure 2.**
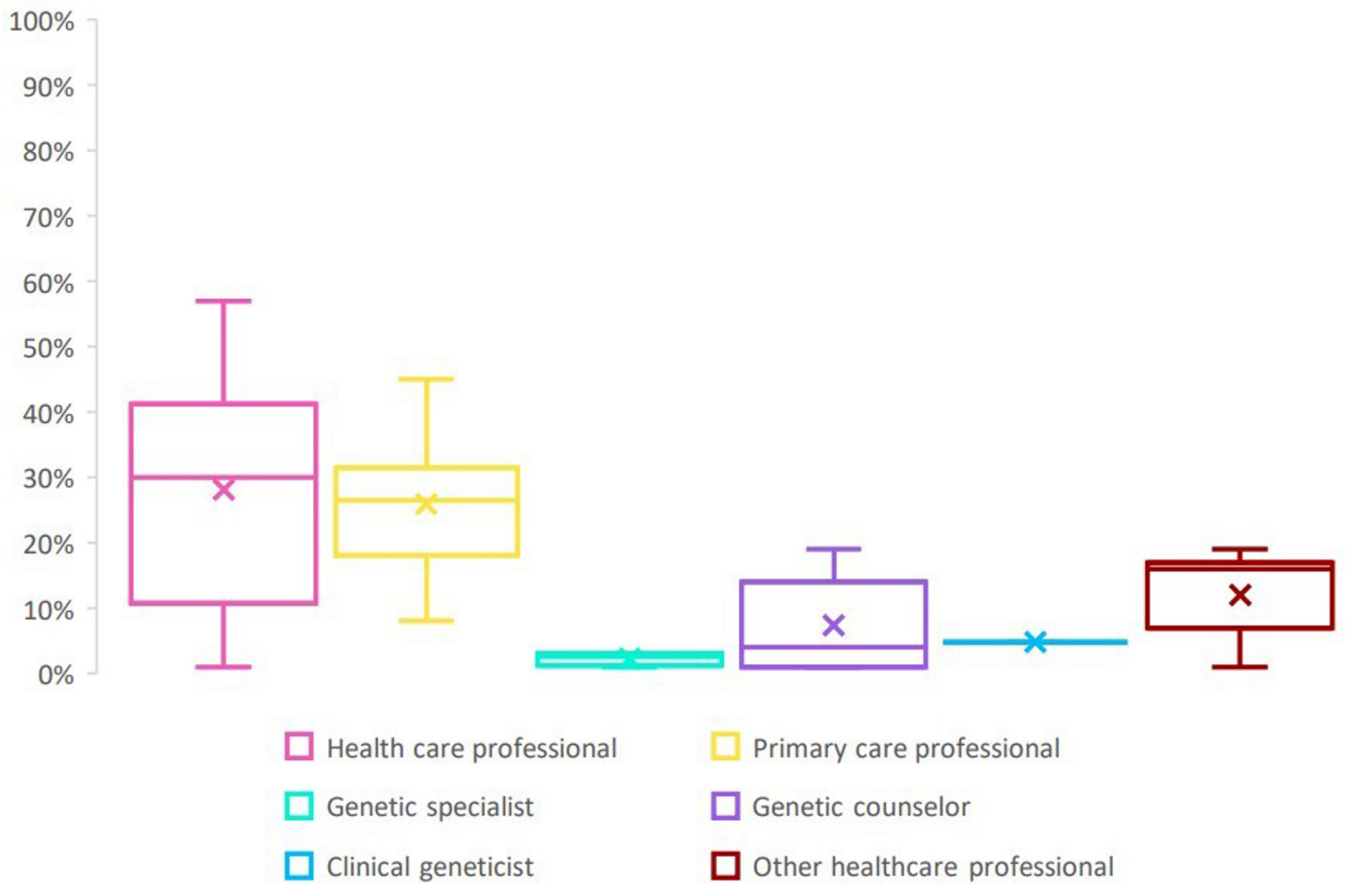
Sharing behavior of DTC-GT consumers with health care professionals.

A more detailed analysis of the data on sharing behavior with specific healthcare professionals revealed that DTC-GT consumers most commonly reported sharing their results with a primary healthcare professional, with the proportion ranging from 8% to 78% ^33, 34, 36, 38, 39, 41, 44– 47, 49– 51, 54, 56^. Interestingly, those that received a subsidized or free DTC-GT more often shared their DTC-GT with a primary health care professionals (range 27%–78%) ^34, 38, 39, 41, 44– 46, 56^ compared to those that paid out of pocket (8–25%) ^33, 36, 47, 49– 51, 54^. To a lesser extent, data were shared with a trained genetic professional (2–3%) ^41, 44, 45, 49^ like a genetic counselor (range 0.4–19%) ^33– 36, 38, 46, 47, 50, 54^ or a clinical geneticist (0.4–5%) ^36, 50, 54^. Participants in the Scripps Genomic Health Initiative, where genetic counseling was provided at no cost by a Navigenics counselor and proactive outreach was conducted based on their test results, reported a slightly higher frequency of sharing their results with a genetic counselor compared to participants in other studies that specifically examined sharing behavior towards trained genetic professionals ^34, 35, 71^ (see Table 3A) (underlying data). Finally, some DTC-GT consumers also reported to have shared their DTC-GT results with other health care professionals (e.g. oncologist, nurse practitioner, etc.) (range 1–19%) ^33, 36, 41, 44– 47, 49, 50, 52, 54^ (see Figure 2).

**Motivations behind sharing behavior of DTC-GT consumers**

Only a small proportion of the primary studies we identified examined the actual motivations behind DTC-GT consumers' sharing behavior (n=4/28) ^31, 35, 44, 47^. In the Scripps Genomic Health Initiative study, participants who were given the opportunity to consult a Navigenics counselor free of charge provided several reasons for utilizing the offered service ^35^. These included the desire to take advantage of a free service, seeking additional information on risk calculations, the fact that they were actively contacted by a genetic counselor, seeking guidance on managing their health, the chance to discuss family history, and addressing a perceived lack of understanding regarding their genetic results. On the other hand, those who chose not to utilize the free genetic counseling service often felt they already had a clear understanding of their results and did not perceive a need for further explanation, or they planned to consult with the genetic counselor in the future ^35^. Similar patterns were observed in a study by Gordon *et al.* (2012), where some DTC-GT consumers shared their results with their general practitioner in order to gain assistance with the interpretation of their DTC-GT results or to receive advice on how to reduce their risk, while others informed their general practitioner without expecting any follow-up or further testing. Other participants chose not to share their results, as they considered the information to be of limited relevance to their healthcare provider ^31^. Another study by van der Wouden *et al.* (2016), reported that while many participants who discussed their DTC-GT results with primary care professionals or other health care professionals cited health improvement as a key motivation, a substantial proportion expressed concerns about the potential inclusion of genetic results in their medical records ^44^. Furthermore, 42% of participants did not consider their results important enough to share, while 38% intended to discuss their results with a health care professional but had not yet done so due to time constraints ^44^. Finally, in a study by Wang *et al.* (2017), the likelihood of sharing DTC-GT results with medical professionals was influenced by the consumers' reasons for using third-party interpretation services. Health-related motivations to use third-party interpretation services, including concerns about individual and family health, were found to be significantly associated with an increased likelihood of sharing results with healthcare professionals compared to ancestry-related motivations ^47^.

**Experiences of health care professionals with DTC-GT consumers sharing their test results**

Our review identified thirteen studies that have explored the experiences of healthcare professionals regarding patients who disclosed their DTC-GT results ^29, 57– 68^ (see Table 3B) (underlying data). The proportion of health care professionals that had been asked questions about DTC-GT varied, ranging from 19% to 76%. In a study by Goddard *et al.* (2007) 7% of primary care physicians and pediatricians aware of DTC-GT reported having at least one patient who discussed their DTC-GT results with them ^29^. Similar low percentages (3–17%) were observed in other studies conducted between 2010 and 2012 ^57– 61^. In contrast, another study performed by Howard and Borry in 2013 found that 44% of European clinical geneticists (n=54/121) had seen at least one DTC-GT consumer for the sole purpose of reviewing DTC-GT results ^62^. Most of these participants had been contacted by one to five patients with DTC-GT results ^62^. A more recent American study revealed that 40% of genetic counselors had seen at least one DTC-GT consumer specifically for reviewing results ^67^. Likewise, a study assessing physicians’ (primary and specialty care) experiences reported that 35% of participants had patients sharing their DTC-GT results within the past year ^63^. Another American study, focusing on the experiences of genetic counselors with clinical cancer genetics as primary specialty, reported that 94% of study participants had encountered health-related DTC-GT results, and 69% had reviewed health-related third-party interpretation data ^66^. More specifically, these study participants provided counseling for a median of 3 DTC-GT results in the last year ^66^. In addition, McGrath *et al.* (2019) reported that 58% of specialists (genetic counselors and clinical geneticists) had experience with patients bringing their DTC-GT results compared to 17% within the group of primary care healthcare professionals surveyed within their study ^64^ (see Table 3B) (underlying data). Finally, Millward *et al.* (2020) reported that 114 DTC-GT related referrals were received by 11 different Australian genetic services since 2010 ^65^.

**Sharing behavior of DTC-GT consumers with family members**

**Figure 3.**
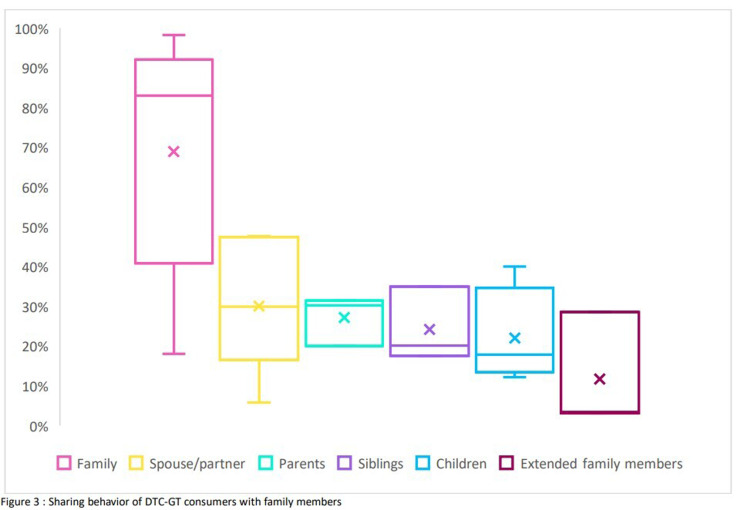
Sharing behavior of DTC-GT consumers with family members.

Our search also identified twelve studies that assessed to what extent DTC-GT consumers had shared their genetic test results with their family members, with the number of participants ranging from 20 to 29478 ^32, 34, 36, 37, 39, 43, 47, 49, 50, 54, 56, 72^ (see Table 4) (Underlying data). The percentage of participants sharing their DTC-GT test with family members ranged from 18% ^32^ to 98% ^53^. More detailed analysis indicated that this was frequently the case with partners (6%–48%) ^32, 36, 43, 48, 50, 54^, parents (20%–31%) ^36, 50, 54^, and siblings (18%–35%) ^36, 50, 54^. Sharing of test results with extended family members occurred less frequently (3%–29%) ^36, 50, 54^ (see Figure 3).

***B. Health care utilization***

Overall, we identified 13 studies assessing healthcare utilization following DTC-GT, with a particular focus on the validation of DTC-GT results and subsequent clinical actions. An early study by Powel *et al.* (2012) found that four out of five primary care physicians who encountered patients bringing in DTC-GT results did no alter their patient’s medical management ^61^. However, other studies did report on several instances of DTC-GT result validation and clinical actions performed based on the DTC-GT findings. Krieger *et al*. (2016) reported that 9% (n=55/617) of participants stated to have received additional genetic tests, medical exams, screenings, procedures, and/or scans as a result of the genetic information they had received through DTC-GT ^42^. A similar result was reported by Lee *et al.* (2020) in a web-based survey among adult adoptees that completed DTC-GT. In this study 18% (n=5) of respondents received additional testing or procedures based on DTC-GT results ^53^. A qualitative interview study by Francke *et al.* (2013) examined the experiences of 11 women who tested positive for BRCA through DTC-GT. Among them, one had undergone a prophylactic mastectomy, while three others had planned the procedure. Additionally, three had undergone oophorectomies, and four had planned to do so after completing childbearing. Five participants sought breast exams and imaging following their results, while seven participants who neither had nor planned to undergo mastectomies reported continuing regular breast cancer monitoring. Furthermore, 30 secondary BRCA tests were conducted on family members as a result of the initial DTC-GT, either through a health care professional or 23andMe, yielding 13 positive and 17 negative results. In one mutation-positive secondary case, early-stage breast cancer was detected. Several family members with BRCA-positive results had already undergone prophylactic mastectomy and oophorectomy, while others indicated plans to pursue these risk-reducing procedures in the future ^36^.

Jonas *et al.* (2019) reported that 40% of healthcare professionals (primary and specialty care physicians) who received DTC-GT results from patients made referrals to other healthcare professionals in the preceding year. Among these referrals, 78% were made to clinical geneticists and genetic counselors, while 22% were made to other health care professionals (e.g. clinical pharmacists) ^63^. Trained genetic professionals also reported to have referred patients to other health care professionals in the study by Giovanni *et al.* (2010). In this study, 15 specialists in clinical genetics (n=15/22, 68%) had referred patients to at least one other health care professional ^57^. In the Australian study conducted by Millward *et al.* (2020), which examined clinical genetics service referrals related to DTC-GT over ten years (2010–2019), there was considerable variation in the number of DTC-GT related referrals across the different publicly funded services surveyed ^65^. While most services lacked specific procedures/guidelines for managing DTC-GT related referrals, six services reported that validating DTC-GT results was the most frequently performed clinical action following an appointment ^65^. Overall, these services estimated that they had attempted to validate DTC-GT results for 34 patients, of which only 3 (9%) were confirmed as correct ^65^. In the PGen study, 11% (n=105/961) of DTC-GT consumers stated to have undergone follow-up tests, examinations, and procedures based on their DTC-GT results six months after obtaining their test results ^45^. A similar finding was also reported by Kaufman *et al.* (2012) where on average 10% of DTC-GT consumers reported following up their results with additional laboratory tests. This percentage was higher among those participants who had shared their DTC-GT test results with a health care professional (26%) compared to those who did not (2%) ^33^. Interestingly, 27% of participants in the SGHI research study reported that their physicians had ordered additional tests based on the DTC-GT results they received ^34^. Another American study by Elson *et al.* (2020) explored the outcomes of receiving DTC-GT results for the two most common genetic risk factors for venous thromboembolism (Factor V Leiden and prothrombin 20210G>A). In this study, 21% of variant-positive individuals were advised by health care professionals to undergo repeat genetic testing in a clinical lab, 13% were advised to have additional testing for other clotting disorders, and 15% were recommended to have relatives tested. About 28% (n=82/294) of cases reported that their relatives also underwent genetic testing based on their DTC-GT results. Within this group, 34% (n=28/82) reported that their family members received genetic testing through a healthcare professional and 17% (n=14/82) through a combination of DTC-GT and a health care professional ^50^. In another study by Koop *et al.* (2021), six participants indicated to have pursued evaluation with a specialist in hereditary hemochromatosis (HH) due to their positive DTC-GT result. Additionally, three participants saw a HH specialist because a family member tested positive on commercial DNA testing for HH. Three participants also stated that a primary care physician ordered additional genetic testing due to a positive DTC-GT result, with one participant receiving conflicting results from formal gene testing ^52^. Finally, in the study by Ashenhurst *et al.* (2022), 26.4% of participants with a physician-diagnosed AATD received their diagnosis only after receiving their 23andMe test results and 50% of individuals with a PI*ZZ genotype (identified through DTC-GT) had received a diagnosis of AATD by a physician ^54^.

## Discussion

The findings of this systematic review offer a detailed synthesis of the sharing behavior and healthcare utilization following DTC-GT. Compared to the previous review by Stewart *et al.* (2017), which identified 14 articles on the sharing behavior of DTC-GT consumers, our review identified 40 articles which specifically examined the sharing behavior of actual DTC-GT consumers, experiences of health care professionals with DTC-GT consumers sharing their test results and/or healthcare utilization following DTC-GT ^25^. Our analysis indicates that a considerable number of individuals who underwent DTC-GT have disclosed their test results to both healthcare professionals and family members. The proportion of DTC-GT consumers who report to have shared their DTC-GT results with healthcare professionals has remained relatively stable in recent years. However, the likelihood of sharing these results seems to be influenced, to some extent, by the cost of the test. Specifically, those who received subsidized or free tests were more likely to share their results, particularly to primary healthcare professionals, compared to those who paid out-of-pocket. This suggests that individuals who pay out-of-pocket for DTC-GT tests may be less motivated to engage with healthcare professionals. Surprisingly, only four of the primary studies we identified assessed the motivations behind consumers’ decisions to share their DTC-GT results ^31, 35, 44, 47^. The choice to either share or withhold DTC-GT results from healthcare professionals appears to be driven by a variety of motivational factors. Given that not all instances of sharing DTC-GT results will automatically lead to increased medical utilization, as was also demonstrated by the included studies, it is important to gain a better understanding of the underlying motivations of DTC-GT consumers for sharing their test results.

While not all DTC-GT consumers shared their test results with healthcare professionals, there is a notable trend of increasing interactions between DTC-GT consumers and health care professionals. This is particularly visible in studies assessing the experiences of genetic health professionals. For example, data from a 2017 survey of genetic counselors active in the US reveal that 40% had seen at least one consumer in the clinic solely for the purpose of reviewing DTC-GT test results, and 76% had been queried about DTC-GT by at least one patient ^67^. This represents a significant rise from an earlier survey performed in 2008, where only 14% of genetic counselors reported receiving such requests ^58^. This increase could be explained by the growing number of actual DTC-GT consumers. Considering the ongoing growth in the DTC-GT industry, it is likely that the impact of DTC-GT on healthcare systems will continue to increase in the future ^67^. As more DTC-GT companies continue to develop partnerships with physician intermediaries in the ordering and test reporting process, patients may also potentially turn to these professionals for additional health information and advice ^63^. Findings from the Scripps Genomic Health Initiative indicate that offering free genetic counseling and proactive outreach organized by DTC-GT companies could increase the likelihood of DTC-GT consumers discussing their DTC-GT results with a trained genetic professional ^34, 35, 38^. This highlights the importance of facilitating proactive post-test communication through trained healthcare professionals employed by DTC-GT companies, which could help consumers better understand their results and potentially reduce the need for (unnecessary) follow-up consultations within the healthcare system ^30, 67^. By prioritizing ethical and consumer-focused practices, DTC-GT companies could enhance transparency, improve accessibility, and build trust in their services.

The impact of DTC-GT on healthcare systems has been a subject of considerable debate ^57, 62^. Initial concerns about DTC-GT suggested that it could lead to a significant burden on healthcare systems, including increased downstream tests, procedures, and referrals to specialists. Many of the anticipated issues have not manifested as expected based on current findings. It seems that previous studies that assessed sharing behavior more hypothetically have overestimated the proportion of consumers that would share DTC-GT results with a health care professional ^33^. Most recent study findings indicate that patients sharing results from DTC-GT only constitute a small proportion of the total amount of patient visits in clinical (genetics) services ^63, 65^. Nevertheless, clinical actions (e.g. testing of family members) were taken for some DTC-GT related referrals clinical actions, which also demonstrates the impact that DTC-GT could have on the health care system ^65^. The ongoing shift from testing for monogenic disorders to screening for polygenic conditions could also further impact the healthcare system, as polygenic risk scores add complexity, making interpretation more challenging for both consumers and healthcare professionals ^73, 74^. As genetic information becomes more complex, more individuals may seek professional guidance, potentially increasing demand for healthcare services and straining healthcare resources. Unlike monogenic conditions, where genetic variants typically have well-established clinical significance, polygenic risk scores remain less straightforward to interpret and validate. Without standardized guidelines and sufficient resources, inconsistent interpretation could again lead to unnecessary consultations and inefficient use of healthcare services.

A recent Australian study investigating the impact of DTC-GT on clinical genetics services revealed that while most referrals resulted in patient appointments, the willingness of services to offer appointments varied ^65^. Healthcare professionals have also voiced to feel pressured to refer patients for clinical confirmatory testing of DTC-GT results and/or additional screening/testing, especially considering the evidence of the high error rate of DTC-GT. A recent American study by Tandy-Connor *et al.* (2018) found that 63% (31/49) of patients that were seeking confirmatory testing of their obtained raw data through third-party interpretation services did not receive important genetic health risk information within their original DTC-GT report ^75^. In the same study, it was also discovered that 40% of the reported variants across a range of patient samples turned out to be false positives ^75^. Results were even more concerning in the survey study among Australian clinical genetics services, where fewer than 10% of variants were confirmed among DTC-GT referrals for consumers who had used third-party interpretation services ^65^. The need for confirmatory testing to prevent inappropriate patient care and medical management could present significant challenges in healthcare systems facing financial constraints and systemic barriers, such as limited access to specialized care or testing services. Furthermore, appointments involving DTC-GT consumers may also redirect healthcare resources away from patients with a clear clinical indication which might present challenges to ensure equitable healthcare delivery ^12^. In response to these issues, the Royal College of General Practitioners and the British Society for Genetic Medicine issued a position statement in 2019 advising against referrals to clinical genetics services solely based on DTC-GT results. Instead, they recommend conducting a thorough risk assessment and evaluating family medical history prior to considering referrals in accordance with standard clinical pathways and protocols ^76^. This differs from the position statement issued by the National Society of Genetic Counselors (NSGC) which recommend that “Results obtained through at-home genetic tests should be reviewed with a genetics specialist, as some findings may need to be confirmed in a clinical laboratory before being used in healthcare decision-making” ^77^.

Interestingly, our findings indicate that DTC-GT consumers more often shared their results with primary health care professionals (e.g. GP) rather than with trained genetic professionals ^46^. Prior research has demonstrated that primary healthcare professionals lack time and feel unprepared or unqualified to take on the gatekeeping role expected of them when helping their patients navigating DTC-GT results ^59, 69, 78, 79^. A particular proportion of referrals to the Australian clinical genetics services that were surveyed in the study of Millward *et al.* (2020) were made by GPs who were unsure about the significance of the results that their patients had received ^65^. These findings underscore the urgent need for better education and training for GPs and primary care health care professionals in handling genetic information. As primary points of contact in the healthcare system, these health care professionals must be equipped with concrete practice guidelines to guide decisions about referral and follow-up care for those results requiring specialist attention ^46, 63^. In a study by McGrath *et al.* (2019), medical providers without specific training in genetics but with prior experience consulting DTC-GT consumers were more likely to accurately interpret genetic test results compared to those without any prior experience with genetic test consultations ^64^. This improved ability may stem from these providers actively seeking additional information to assist with interpretation and counseling, which ultimately helps them develop and enhance their skills in this area ^64^. This finding suggests that integrating genetics education into the curricula of various (primary) healthcare professionals could be highly beneficial ^64, 79^.

Variations in research designs, outcome measures, and participant characteristics across the identified studies complicate direct comparisons and hinder the ability to draw definitive conclusions. This observed heterogeneity underscores the need for more standardized approaches. Conducting further international studies with consistent outcome measures would be highly beneficial in gaining deeper insights into the broader impact of DTC-GT on healthcare systems. Such research would not only enable more meaningful comparisons but also contribute to a clearer understanding of DTC-GT's implications across diverse healthcare contexts. The current body of literature remains heavily focused on the U.S. context, with limited exploration of sharing behavior and health care utilization following DTC-GT in other regions of the world. As healthcare systems, cultural norms, and healthcare access vary greatly across regions, conducting studies in diverse international contexts would provide a more comprehensive view of the global implications of DTC-GT. 

## Conclusion

The findings of this systematic review provide a comprehensive overview of the current scientific evidence on the sharing behaviors and healthcare utilization patterns following DTC-GT. While initial concerns about DTC-GT's impact on the health care system have not fully materialized, the increasing number of consumers engaging with healthcare professionals underscores the need for preparedness and appropriate policy responses. Policymakers and healthcare institutions must also take proactive measures to equip health care professionals—particularly those in primary care—with the knowledge and tools necessary to navigate the evolving challenges associated with DTC-GT. Given that general practitioners are often the first point of contact for DTC-GT consumers, enhancing their genetic literacy through targeted training programs and decision-support tools could help mitigate the risk of unnecessary referrals, misinterpretation of results, and inefficient healthcare utilization. As the majority of existing literature focuses on high-income countries, significant gaps remain in understanding how DTC-GT is utilized in regions with differing healthcare infrastructures, regulatory frameworks, and access to genetic services. Future research should therefore prioritize standardizing study methodologies and expanding investigations beyond the U.S. context to better capture the global impact of DTC-GT.

## Ethics and consent

No ethics and consent were required.

## Data Availability

KU Leuven RDR (Dataverse): Data related to the manuscript: Sharing behavior and health care utilization following direct-to-consumer genetic testing: a systematic review. https://doi.org/10.48804/EWC2MA ^80^ This project contains the following underlying data: -Figure1 PRISMA.pdf -PRISMA_2020_abstract_checklist.pdf - README file _DTCGT Review.txt -Supplementary materials.pdf -Table 1_DTCGTConsumer.pdf -Table2_HCP.pdf -Table3_Sharingbehavior.pdf -Table4_Family.pdf Data are available under the terms of the Creative Commons Attribution 4.0 International license (CC-BY 4.0) ( https://creativecommons.org/licenses/by/4.0/). KU Leuven RDR (Dataverse): PRISMA checklist for ‘Sharing behavior and health care utilization following direct-to-consumer genetic testing: a systematic review’. https://doi.org/10.48804/EWC2MA ^80^ Data are available under the terms of the Creative Commons Attribution 4.0 International license (CC-BY 4.0) ( https://creativecommons.org/licenses/by/4.0/).
