# Supplementary material for: Sharing behavior and health care utilization following direct-to-consumer genetic testing: a systematic review
Source: Open Res Eur. 2025 Sep 19;5:74. Originally published 2025 Mar 21. [Version 2] doi: 10.12688/openreseurope.19751.2 (PMC12770896; doi:10.12688/openreseurope.19751.2)
Supplement: Peer review history

# Reviewer report: Reviewer response for version 2

**DOI:** 10.21956/openreseurope.23058.r65290

**Published:** 2026-01-05

**Authors:** Diane C. Seibert

**Affiliations:** 1 Uniformed Services University of the Health Sciences, Bethesda, Maryland, USA

Interesting and useful article --- and I appreciate (changes made in response to comments made by the 1st reviewers (MeSH terms), etc. 

 My only comments were about formatting -- all are are on pages 7 & 8.  Please  review all the times you refer the reader to a Table and then mention 'underlying data'.  I'm not sure what you mean by 'underlying data'....that's what I'm going to a table to review, no??  You could just delete those 2 words, and it would be fine.  BUT if you leave them in, please be consistent in your use of parentheses and case (upper/lower).  Also, in the paragraph beginning with "Sharing behavior ofDTC-GT consumers with family members, please review the use 2nd to last sentence and the contents in the final parentheses.  I'm not sure what the 18%-35% revers to ...median perhaps?

 Otherwise, I think it's ready to index.

Are the rationale for, and objectives of, the Systematic Review clearly stated?

Yes

Is the statistical analysis and its interpretation appropriate?

Yes

Are sufficient details of the methods and analysis provided to allow replication by others?

Yes

Are the conclusions drawn adequately supported by the results presented in the review?

Yes

Reviewer Expertise:

20+ year history of educating health professionals (Nurse Practitioners genomic literacy, fluency and competency) has been my focus.

I confirm that I have read this submission and believe that I have an appropriate level of expertise to confirm that it is of an acceptable scientific standard.

# Reviewer report: Reviewer response for version 2

**DOI:** 10.21956/openreseurope.23058.r60661

**Published:** 2025-11-06

**Authors:** Kurt Christensen

**Affiliations:** 1 Department of Population Medicine, Harvard Pilgrim Health Care Institute and Harvard Medical School, Boston, Massachusetts, USA

**Review of Revision to “Sharing Behavior and Health Care Utilization Following Direct-to-Consumer Genetic Testing: A Systematic Review”**

 The authors have done a commendable job addressing concerns raised during the initial review. I have only a few remaining issues:

**Treatment of Duplicate Data Reporting:**

 The handling of repeated reporting of the same results remains problematic. While the authors have been transparent in their analytic approach, it is clear that citations 40, 41, and 44 from the PGen Study report data from the same participant cohort, not distinct subgroups. Variations in sharing percentages stem from differing analytic strategies (e.g., handling of missing data), not from subgroup differences. Treating these findings as independent data points artificially triples the weight of a single study in the boxplots. I recommend omitting data from citations 40 and 41 when constructing the boxplots. Additionally, using boxplots that assign equal weight to studies with 20 participants and those with over 30,000 is methodologically questionable.

**Discussion of Study Design Differences:**

 The Discussion section should address differences in DTC recipients and study designs as potential sources of data discrepancies. For example, the study reporting only 1% of participants sharing results with clinicians was the Multiplex Initiative, which (a) excluded patients with any of the eight common conditions examined by the genetic test, and (b) conducted follow-up only three months post-disclosure. The combination of a very healthy cohort and short follow-up likely explains the low clinician-sharing rate.

**Clarification of the Multiplex Initiative’s Nature:**

 Technically, the Multiplex Initiative was a clinical service offered to insured patients of the Henry Ford Health System. However, its methodology was intentionally designed to mirror the processes of DTC companies wherever feasible

Are the rationale for, and objectives of, the Systematic Review clearly stated?

Yes

Is the statistical analysis and its interpretation appropriate?

Partly

Are sufficient details of the methods and analysis provided to allow replication by others?

Partly

Are the conclusions drawn adequately supported by the results presented in the review?

Partly

Reviewer Expertise:

Medical, behavioral, and economic impact of genomic testing of healthy individuals

I confirm that I have read this submission and believe that I have an appropriate level of expertise to confirm that it is of an acceptable scientific standard, however I have significant reservations, as outlined above.

# Reviewer report: Reviewer response for version 1

**DOI:** 10.21956/openreseurope.21365.r53486

**Published:** 2025-06-02

**Authors:** Lola Cook

**Affiliations:** 1 Indiana University Bloomington, Bloomington, Indiana, USA

This article is a systematic review of sharing behavior and health care usage after genetic testing through a direct-to-consumer company. It is a timely article with important points and well written. It builds upon prior evidence.

 Major comments:

 Authors should address overall quality of evidence in the body of the text so the reader can easily refer to and understand any gaps; also though they use the Hawker method to assess quality this does not include addressing any bias within the literature. Most of the numerical analysis in the text involves ranges across studies that are often large and thus, somewhat meaningless, (i.e.1% to 57% is given as the proportion who share their test results with a HCP) and do not necessarily support their conclusions in the Discussion such as "Our analysis indicates that a considerable number of individuals who underwent DTC-GT have disclosed their test results to both healthcare professionals and family members". When they discuss the detailed results, specifically these large ranges, they refer to the box plot figures without further characterization of the data displayed. More details regarding how the data are distributed would be helpful and perhaps support some of the conclusions. They correctly compare ranges across categories. Also lacking are summarizing key demographic features of respondents such as level of education which could impact results and conclusions. An overview of the types of results reported would also be informative. There are additional limitations of the data not reported such as few articles about family sharing broke down categories of family members, for example, so please note any gaps or deficiencies in the literature your reviewed. For example, it is noted that the data tend to be US-centric.

 Minor comments:

 Check for typos throughout article including in Abstract. 

 Define AATD. 

 This statement is confusing: "Participants in the Scripps Genomic Health Initiative, where genetic counseling was provided at no cost by a Navigenics counselor and proactive outreach was conducted based on their test results, reported a slightly higher frequency of sharing their results with a genetic counselor compared to participants in other studies" since it is not clear that the sharing reported is with the counselor at the DTC company or external.

Are the rationale for, and objectives of, the Systematic Review clearly stated?

Yes

Is the statistical analysis and its interpretation appropriate?

Yes

Are sufficient details of the methods and analysis provided to allow replication by others?

Yes

Are the conclusions drawn adequately supported by the results presented in the review?

Partly

Reviewer Expertise:

genetic counseling, genetic testing, Parkinson disease

I confirm that I have read this submission and believe that I have an appropriate level of expertise to confirm that it is of an acceptable scientific standard, however I have significant reservations, as outlined above.

# Response

**Published:** 2025-08-29

**Authors:** eva Van Steijvoort

**Affiliations:** Public Health and Primary Care, KU Leuven, Leuven, Vlaams-Brabant, Belgium

We appreciate your careful review of our manuscript and the detailed, constructive feedback provided. Your comments have helped clarify our analyses, highlight limitations more explicitly, and improve the overall presentation of the work. Below, we respond to each of the points raised. Major comments:

- Authors should address overall quality of evidence in the body of the text so the reader can easily refer to and understand any gaps; also though they use the Hawker method to assess quality this does not include addressing any bias within the literature.

We would like to clarify that the Hawker method does incorporate evaluation of ethical considerations and potential sources of bias within its appraisal criteria, and these aspects were systematically assessed for all studies included in our review. To ensure transparency, we have integrated the following paragraph into the body of the text. *“The quality appraisal of the included studies, conducted using Hawker et al.’s (2002) framework, revealed that most articles were generally of good quality across key domains such as titles and abstracts, introductions, methodology, data analysis, results, and implications. However, recurring limitations emerged in areas related to sampling, ethics, and bias, as well as transferability or generalizability, where many studies were rated only “fair” or occasionally “poor.” Despite these weaknesses, nearly all studies were judged to present findings that were useful and relevant to the research questions.”*

- Most of the numerical analysis in the text involves ranges across studies that are often large and thus, somewhat meaningless, (i.e.1% to 57% is given as the proportion who share their test results with a HCP) and do not necessarily support their conclusions in the Discussion such as "Our analysis indicates that a considerable number of individuals who underwent DTC-GT have disclosed their test results to both healthcare professionals and family members". When they discuss the detailed results, specifically these large ranges, they refer to the box plot figures without further characterization of the data displayed. More details regarding how the data are distributed would be helpful and perhaps support some of the conclusions. They correctly compare ranges across categories.

We recognize that the reported ranges across studies are wide, reflecting considerable variability in the primary data. To ensure comprehensive transparency, all underlying data and study-specific results, including sample sizes and contextual details, are provided in the accompanying tables. In response to this concern, we have enhanced the boxplot figures by including more detailed information to better depict the data distribution. Furthermore, the manuscript text has been expanded to describe these features more thoroughly and offer clearer context for interpreting the figures. We believe these improvements increase clarity about the distribution of the data and strengthen the support for our conclusions.

- Also lacking are summarizing key demographic features of respondents such as level of education which could impact results and conclusions.

We would like to underline that this information is available to the reader in Table 1.

- An overview of the types of results reported would also be informative.

We would like to emphasize that the data provided in the primary studies do not permit such detailed analysis and reporting. In many cases, it is unclear which specific types of results participants received. When available, we have included details of the company and test used in Table 1; however, we acknowledge that this information remains limited.

- There are additional limitations of the data not reported such as few articles about family sharing broke down categories of family members, for example, so please note any gaps or deficiencies in the literature your reviewed. For example, it is noted that the data tend to be US-centric.

We would like to direct your attention to the final paragraph of our discussion section, where we address these concerns. We highlight the considerable variation and heterogeneity in study designs and measures that complicate direct comparisons, emphasizing the need for more standardized approaches in future research. Additionally, we discuss the current U.S.-centric focus of the literature and underscore the importance of conducting more international studies to better capture diverse healthcare systems and cultural contexts. *“Variations in research designs, outcome measures, and participant characteristics across the identified studies complicate direct comparisons and hinder the ability to draw definitive conclusions. This observed heterogeneity underscores the need for more standardized approaches. Conducting further international studies with consistent outcome measures would be highly beneficial in gaining deeper insights into the broader impact of DTC-GT on healthcare systems. Such research would not only enable more meaningful comparisons but also contribute to a clearer understanding of DTC-GT's implications across diverse healthcare contexts. The current body of literature remains heavily focused on the U.S. context, with limited exploration of sharing behavior and health care utilization following DTC-GT in other regions of the world. As healthcare systems, cultural norms, and healthcare access vary greatly across regions, conducting studies in diverse international contexts would provide a more comprehensive view of the global implications of DTC-GT.”* Minor comments:

- Check for typos throughout article including in Abstract.

We have carefully checked the entire article, including the Abstract, for any typos and corrected them accordingly.

- Define AATD.

The definition of AATD has now been added to the manuscript for clarity.

- This statement is confusing: "Participants in the Scripps Genomic Health Initiative, where genetic counseling was provided at no cost by a Navigenics counselor and proactive outreach was conducted based on their test results, reported a slightly higher frequency of sharing their results with a genetic counselor compared to participants in other studies" since it is not clear that the sharing reported is with the counselor at the DTC company or external.

We understand the confusion. While this information was included in Table 3A, we have now also clarified in the manuscript that the sharing reported was specifically with a Navigenics counselor.

# Reviewer report: Reviewer response for version 1

**DOI:** 10.21956/openreseurope.21365.r53178

**Published:** 2025-05-07

**Authors:** Kurt Christensen

**Affiliations:** 1 Department of Population Medicine, Harvard Pilgrim Health Care Institute and Harvard Medical School, Boston, Massachusetts, USA

This manuscript reported findings from a systematic review of studies of information sharing and health care utilization following direct-to-consumer genetic testing. The article is generally well-written, particularly in the Discussion section which presents thoughtful recommendations to address data gaps and methodological inconsistencies, as well as challenges for primary care professionals and other clinicians. I’m uncertain about how well some arguments are supported, however, due to uncertainties about how data in the examined studies were analyzed and due to instances where the authors did not note important differences testing approaches or study populations. The manuscript would benefit from addressing these following concerns:

  

- The authors note that six articles they analyzed were products of the PGen study and three articles were products of the Scripps Genomic Health Initiative. How did the authors address the potential for double-counting sharing data and health care utilization data?
- Adoptees are a focus of a few articles, including the one where 98% of study participants reported sharing results with family members. The manuscript would benefit from greater sensitivity to studies that focused on particular populations and study designs that deliberately tried to offer to DTC-GT to populations who may not have been motivated to pursue testing (e.g., the Multiplex Initiative).
- The introduction is appropriately sensitive to differences between health-related and non-health-related DTC-GTs, as well as the differences in the types of health information provided by health-related DTC-GTs. Yet, the review’s analyses don’t seem sensitive to these important differences. Analyses of sharing with health care providers and health care utilization greater sensitivity to studies where health-relevant information was unlikely to be generated (e.g. ancestry-focused testing) and, conversely, studies where information sharing may be more likely (e.g., studies where testing provided carrier status).

Other Important Concerns

 I did not see any key articles missing, but I encourage the authors to include “Direct-To-Consumer Screening and Testing,” “Direct-To-Consumer Screening” and “Direct-To-Consumer Testing” in their search strings given that these are the preferred MeSH entries (perhaps intersecting with the MeSH term, “genetic testing”).

 The time frame of the analyses is a little hard to identify for each study and may have a strong association with the likelihood of information sharing and medical follow-up.

 In analyses of data from the Scripps Genomic Health Initiative, health care utilization was assessed as follows: “13 health screening behaviours were assessed by asking

 about actual completion of a given screening test…” Are the authors sure that they’re summarizing data about health care utilization motivated by DTC-GT findings? Some of those services (e.g., glucose exam, blood test, mammogram) are common for patients as part of overall preventive care.

 Minor Issues

 The manuscript starts with a focus on monogenic disease risks and risk predictions and feels out-of-focus with the majority of their analyses which address broader range of DTC-GTs.

 In the Introduction, the authors write, “The reliability of a positive test or increased risk result after DTC-GT remains limited, as the development of many genetic conditions is also influenced by other factors such as the environment and lifestyle choices.” I encourage the authors to address some of the challenges they address later in the manuscript related to high rates of analytic false positives, as well as concerns about potential misinterpretations of uninformative findings as negatives for genetic risk factors.

 In Table 3A, the cell “Shared with HCP (=yes)” looks like it should have slightly different content and formatting.

 Figures 2 and 3 would benefit from captions. I was unsure if lines represented medians across studies and x’s represent means. Also, some categories (genetic specialist, genetic counselor, medical geneticist) are not mutually exclusive.

 In one place, those authors write that the Stewart paper was published in 2017.

 The authors write, “Specifically, those who received subsidized or free tests were more likely to share their results, particularly to primary healthcare professionals, compared to those who paid out-of-pocket.” It may be helpful to understand whether those receiving subsidized or free tests were studies where testing was encouraged by researchers, and to note that individuals often pursue DTC-GT to avoid having results put in their medical records, as noted by the authors.

 I don’t think the literature about the use of 3 ^rd^ party interpretation services is very relevant in the Introduction or Discussion, although I agree that the use of these services following DTC-GT is of great concern.

Are the rationale for, and objectives of, the Systematic Review clearly stated?

Yes

Is the statistical analysis and its interpretation appropriate?

Partly

Are sufficient details of the methods and analysis provided to allow replication by others?

Partly

Are the conclusions drawn adequately supported by the results presented in the review?

Partly

Reviewer Expertise:

Medical, behavioral, and economic impact of genomic testing of healthy individuals

I confirm that I have read this submission and believe that I have an appropriate level of expertise to confirm that it is of an acceptable scientific standard, however I have significant reservations, as outlined above.

# Response

**Published:** 2025-08-29

**Authors:** eva Van Steijvoort

**Affiliations:** Public Health and Primary Care, KU Leuven, Leuven, Vlaams-Brabant, Belgium

We appreciate your careful review of our manuscript and the detailed, constructive feedback provided. Your comments have helped clarify our analyses, highlight limitations more explicitly, and improve the overall presentation of the work. Below, we respond to each of the points raised.

- The authors note that six articles they analyzed were products of the PGen study and three articles were products of the Scripps Genomic Health Initiative. How did the authors address the potential for double-counting sharing data and health care utilization data?

We appreciate the question regarding the potential for double-counting data in our analysis, particularly given that six of the included articles originated from the PGen Study and three from the Scripps Genomic Health Initiative. We have been transparent about the inclusion of multiple publications stemming from these two projects. This was clearly disclosed in our results sections. While the studies may share an overarching participant base, the individual publications addressed distinct research questions and often reported on participant subgroups. We recognize that there may be some overlap in study populations, which could pose limitations. By being transparent about the origins of the studies included, we enable readers to interpret the findings with appropriate context. We believe this transparency, combined with our careful analysis of each article, supports a methodologically sound and comprehensive synthesis. We have included the following paragraph in the Limitations section of the Discussion to fully and transparently address this issue. “Additionally, some included articles originated from the same research projects (e.g., PGen Study, Scripps Genomic Health Initiative), raising the possibility of overlapping participant data. While each publication addressed distinct research questions or subgroups, some duplication of outcomes—particularly related to data sharing and health care utilization—may have occurred. We mitigated this by analyzing each article independently and clearly reporting the origin of each study to support transparency and allow readers to interpret the findings in context.”

- Adoptees are a focus of a few articles, including the one where 98% of study participants reported sharing results with family members. The manuscript would benefit from greater sensitivity to studies that focused on particular populations and study designs that deliberately tried to offer to DTC-GT to populations who may not have been motivated to pursue testing (e.g., the Multiplex Initiative).

We agree that studies focusing on specific populations—such as adoptees or participants in initiatives like the Multiplex Initiative—offer unique perspectives on engagement with DTC-GT, particularly among individuals who may not have actively sought out testing on their own. Throughout the manuscript, we have been attentive to the focus and context of each included study. We describe the study populations, recruitment strategies, and design features to ensure that readers understand the diversity of approaches and participant experiences represented in the literature. These details are integrated into our results and discussion sections, where we consider how differences in study design and target populations may influence outcomes such as result-sharing behavior or health care utilization. *“For those who received a subsidized test, the sharing rate was between 19% and 39%* *34* *,* *35* *,* *38* *,* *40* *–* *42* *,* *44* *–* *46* *,* *56* *, while participants who received a free test shared their results at a rate ranging from 1% to 42%* *31* *,* *32* *,* *39* *.”* *“Interestingly, those that received a subsidized or free DTC-GT more often shared their DTC-GT with a primary health care professionals (range 27%–78%)* *34* *,* *38* *,* *39* *,* *41* *,* *44* *–* *46* *,* *56* *compared to those that paid out of pocket (8–25%)* *33* *,* *36* *,* *47* *,* *49* *–* *51* *,* *54* *.”* *“… However, the likelihood of sharing these results seems to be influenced, to some extent, by the cost of the test. Specifically, those who received subsidized or free tests were more likely to share their results, particularly to primary healthcare professionals, compared to those who paid out-of-pocket. This suggests that individuals who pay out-of-pocket for DTC-GT tests may be less motivated to engage with healthcare professionals.”*

- The introduction is appropriately sensitive to differences between health-related and non-health-related DTC-GTs, as well as the differences in the types of health information provided by health-related DTC-GTs. Yet, the review’s analyses don’t seem sensitive to these important differences. Analyses of sharing with health care providers and health care utilization greater sensitivity to studies where health-relevant information was unlikely to be generated (e.g. ancestry-focused testing) and, conversely, studies where information sharing may be more likely (e.g., studies where testing provided carrier status).

We agree with the reviewer that such a stratified analysis would be informative and insightful and would add valuable nuance to analyses of information sharing with healthcare providers and subsequent healthcare utilization. However, we would like to emphasize that the data reported in the primary studies do not support such a detailed analysis. In many instances, it is not clear what specific types of results participants received, and few studies distinguish between those who received health-related findings (e.g., carrier status) and those whose results were limited to non-health-related information (e.g., ancestry). Moreover, as discussed in our manuscript, the distinction between health-related and non-health-related DTC-GT results has become increasingly difficult to draw. The widespread availability of third-party interpretation services enables consumers to upload their raw genetic data and receive additional analyses. These services often provide information on genetic variants or risk scores that were not part of the original DTC-GT offering, thereby extending the scope of interpretation beyond what was initially intended. As a result, the line between health-related and non-health-related genetic information is increasingly blurred, complicating research efforts. This complexity limits our ability to meaningfully categorize participants’ experiences based on the original test type and highlights the need for more detailed reporting in future studies to be able to draw any meaningful conclusions. Other Important Concerns I did not see any key articles missing, but I encourage the authors to include “Direct-To-Consumer Screening and Testing,” “Direct-To-Consumer Screening” and “Direct-To-Consumer Testing” in their search strings given that these are the preferred MeSH entries (perhaps intersecting with the MeSH term, “genetic testing”). We would like to thank the reviewer for this suggestion regarding the inclusion of additional MeSH terms such as “Direct-To-Consumer Screening and Testing,” “Direct-To-Consumer Screening,” and “Direct-To-Consumer Testing.” We agree that these are relevant terms and acknowledge their potential value for enhancing comprehensiveness. Our original search strategy, however, employed a broad range of commonly used text-based terms and phrases that align closely with MeSH indexing. As was noted by the reviewer, our current search did not appear to miss any key studies. Given this, and in the interest of maintaining consistency and transparency in our review process, we have elected not to re-run the search at this stage. However, we will make note of this in the limitations and would recommend incorporating these MeSH terms in future replications or updates of the review. The following paragraph was added to the end of the discussion section: *“Our search strategy incorporated a wide range of commonly used keywords and phrases related to direct-to-consumer genetic testing, we did however not include MeSH terms such as “Direct-To-Consumer Screening”. Including these terms may have improved search sensitivity in certain databases. Future updates of this review might incorporate these MeSH terms to enhance the precision and comprehensiveness of the search strategy.”*

 The time frame of the analyses is a little hard to identify for each study and may have a strong association with the likelihood of information sharing and medical follow-up. When available, information regarding the follow-up duration was included in Table 3. However, this information was not reported in all the studies included in our systematic review. We have addressed this limitation by incorporating the following paragraph into the Discussion section: *“Variability in follow-up duration across studies may have influenced the observed rates of information sharing and medical follow-up, as participants with longer follow-up periods may have had more opportunities to act upon the information received. However, detailed information on the exact timing of follow-up was not consistently available across all studies, limiting our ability to adjust for this factor. This should be taken into consideration when interpreting cross-study comparisons.”* In analyses of data from the Scripps Genomic Health Initiative, health care utilization was assessed as follows: “13 health screening behaviours were assessed by asking

 about actual completion of a given screening test…” Are the authors sure that they’re summarizing data about health care utilization motivated by DTC-GT findings? Some of those services (e.g., glucose exam, blood test, mammogram) are common for patients as part of overall preventive care. We understand the concern raised by the reviewer; however, we would like to emphasize that the results reported in our systematic review specifically focus on the validation of DTC-GT results and the subsequent clinical actions taken based on those results. We did not include any screening behaviors that may be part of general preventive care, as described by the reviewer, in our results section. Minor Issues The manuscript starts with a focus on monogenic disease risks and risk predictions and feels out-of-focus with the majority of their analyses which address broader range of DTC-GTs. We appreciate the reviewer’s observation. Our intention in the introductory paragraph was to provide context for the emergence of direct-to-consumer genetic testing (DTC-GT) by outlining the broader evolution of genetic and genomic medicine—from its initial focus on rare monogenic disorders to its expansion into complex disease risk prediction. However, we recognize that this framing may have inadvertently implied a narrower scope than was intended. To address this, we have revised the introduction to more accurately reflect the full range of health-related DTC-GT examined in our review. In the Introduction, the authors write, “The reliability of a positive test or increased risk result after DTC-GT remains limited, as the development of many genetic conditions is also influenced by other factors such as the environment and lifestyle choices.” I encourage the authors to address some of the challenges they address later in the manuscript related to high rates of analytic false positives, as well as concerns about potential misinterpretations of uninformative findings as negatives for genetic risk factors. We have indeed briefly addressed the challenges related to analytic false positives and the potential for misinterpretation of uninformative findings in the manuscript. However, as the primary aim of our review is to assess sharing behaviors, healthcare professional experiences, and healthcare utilization following DTC-GT, an in-depth exploration of these technical and interpretive limitations lies beyond its scope. We believe these issues have been comprehensively addressed in previous literature, and our focus remains on the downstream clinical and behavioral impacts of DTC-GT results. In Table 3A, the cell “Shared with HCP (=yes)” looks like it should have slightly different content and formatting. We are not entirely sure what is meant by the comment regarding the content and formatting of the “Shared with HCP (=yes)” cell in Table 3A. To clarify, this column was not intended to serve as an overarching header for the adjacent columns. In some studies, only a general percentage of participants who shared results with “a healthcare provider” was reported, without further specification of provider type. We hope this explanation clarifies the presentation of the data.

 Figures 2 and 3 would benefit from captions. I was unsure if lines represented medians across studies and x’s represent means. Also, some categories (genetic specialist, genetic counselor, medical geneticist) are not mutually exclusive. We have added a footnote explaining that for the boxplots shown, the line inside the box indicates the median value, whereas the ‘x’ marks the mean. While we acknowledge that these categories are indeed not mutually exclusive, the primary studies included in our analysis varied considerably in how they defined and reported these roles. To maintain consistency and accuracy, we chose to adhere closely to the categorizations as originally presented in the source studies. The following footnote was added to the figures to clarify this: *“Categories are not mutually exclusive but were retained as defined in the original studies for consistency.”* In one place, those authors write that the Stewart paper was published in 2017. Adjusted in the manuscript. The authors write, “Specifically, those who received subsidized or free tests were more likely to share their results, particularly to primary healthcare professionals, compared to those who paid out-of-pocket.” It may be helpful to understand whether those receiving subsidized or free tests were studies where testing was encouraged by researchers, and to note that individuals often pursue DTC-GT to avoid having results put in their medical records, as noted by the authors. We agree that understanding whether individuals who received subsidized or free tests were part of studies where testing was actively encouraged would provide valuable context. Unfortunately, the available data in the included studies do not consistently provide detailed information on the extent of such encouragement or on participants' motivations for pursuing testing. This limits our ability to draw firm conclusions about the relationship between subsidized testing and sharing behaviors. We have interpreted these findings with caution and noted the need for future research to explore how motivations influence result sharing and health care engagement. I don’t think the literature about the use of 3 ^rd^ party interpretation services is very relevant in the Introduction or Discussion, although I agree that the use of these services following DTC-GT is of great concern. We appreciate the concern regarding relevance; however, we believe that discussing these services in the Introduction and Discussion is important for several reasons. Third-party interpretation services have become a significant and growing component of the DTC-GT landscape, influencing how consumers understand and act upon their genetic results. DTC-GT users may turn to these services to seek additional explanation or clinical interpretation that may not be provided directly by DTC companies. This behavior has important implications for how genetic information is shared with family members and health care providers, as well as for subsequent health care utilization. By including this literature, we provide a more comprehensive view of the consumer experience and the broader context in which DTC-GT results are interpreted and acted upon. Omitting this could overlook a key factor shaping downstream behaviors and potential challenges in clinical integration. Therefore, we believe that maintaining the discussion of third-party interpretation services enhances the depth and relevance of our manuscript.
